# Breath Regulation and yogic Exercise An online Therapy for calm and Happiness (BREATH) for frontline hospital and long-term care home staff managing the COVID-19 pandemic: A structured summary of a study protocol for a feasibility study for a randomised controlled trial

**DOI:** 10.1186/s13063-020-04583-w

**Published:** 2020-07-14

**Authors:** Ka Sing Paris Lai, Christine Watt, Emily Ionson, Imants Baruss, Cheryl Forchuk, Javeed Sukhera, Amer M. Burhan, Akshya Vasudev

**Affiliations:** 1 Department of Psychiatry, Western University, 1151 Richmond St, London, ON N6A 3K7 Canada; 2 Department of Psychiatry, Parkwood Institute of Mental Health, 550 Wellington Rd, London, ON N6C 5J1 Canada; 3 Department of Psychology, King’s University College at Western University, 266 Epworth Ave, London, ON N6A 2M3 Canada

**Keywords:** COVID-19, Randomised controlled trial, protocol, Healthcare staff, Work stress, resiliency, mind-body intervention, yoga

## Abstract

**Objectives:**

**Objective 1:** To determine if it is feasible to conduct an RCT of online Sudarshan Kriya Yoga (SKY) for frontline hospital and long-term care home staff under the constraints imposed by the COVID-19 pandemic and need for remote trial monitoring.

**Objective 2:** To assess whether online versions of SKY and/or Health Enhancement Program (HEP) result in improvement in self-rated measures of insomnia, anxiety, depression, and resilience.

**Trial design:**

This is an open-label feasibility randomized controlled trial (RCT), comparing an online breath based yogic intervention SKY versus an online control mind-body intervention HEP in frontline hospital and long-term care home staff managing the COVID-19 pandemic.

**Participants:**

Participants will include frontline hospital and long-term care home staff that are involved in the management of COVID-19 patients in London, Ontario, Canada. Participants will be willing and able to attend via online video conferencing software to participate in the study interventions. Participants must have an adequate understanding of English and be able to sit without physical discomfort for 60 minutes.

**Intervention and comparator:**

Sudarshan Kriya Yoga (SKY): The online version of SKY will be delivered by at least one certified Canadian SKY teacher, with at least one back up teacher at all times, under the supervision of Ms. Ronnie Newman, Director of Research and Health Promotion, Art of Living Foundation, USA. The online version of SKY for healthcare workers has a total duration of 3 hours. Phase I will consist of 5 self-paced online modules of 4-10 minutes each to learn the breath control techniques. Participants will be sent an online survey in REDCap requesting that they self-confirm completion of the Phase I modules. In Phase II, 2 interactive online sessions of 1 hour each will be held on consecutive days with a certified SKY teacher, during which participants will learn the fast, medium and slow breaths. For ease of scheduling, multiple time windows will be offered for Phase II. There will be at least one back up teacher at all times. Both Phase I and II will be completed in the first week.

Health Enhancement Program (HEP): The active control arm, HEP, will consist of time-matched online self-paced modules for Phase I. Phase II will consist of mindfulness-based meditation sessions delivered by mental health staff. HEP will be an active treatment program that incorporates mind-body interventions. HEP will consist of time-matched online self-paced modules with psychoeducation on healthy active living as well as interactive modules comprising of guided de-stressing exercises including music therapy, mindfulness and progressive muscle relaxation. Weekly follow up sessions will be offered to all recruited participants for 30 minutes each for the subsequent 4 weeks in both study arms.

**Main outcomes:**

The following feasibility outcomes will be measured at the end of the study: (1) rate of participant recruitment, (2) rate of retention, (3) completeness of data entry, (4) cost of interventions, and (5) unexpected costs. Such measures will be collected on a daily basis through-out the study and tabulated 5 weeks later at the end of the study.

**Randomisation:**

Participants will be randomized after they have electronically signed the consent form and the research staff have confirmed eligibility. We will use REDCap to perform randomization in a 1:1 ratio as well as allocation concealment. REDCap is widely used by health researchers worldwide to significantly reduce data entry and study management errors to improve data fidelity.

**Blinding (masking):**

All study participants will be blinded to the study hypotheses so as to prevent any expectation bias. Group allocation will be masked during analysis.

**Numbers to be randomised (sample size):**

This study will randomize a total of 60 participants in a 1:1 ratio to either SKY or HEP interventions.

**Trial Status:**

Protocol version number 2.0 (June 5, 2020). Recruitment is currently ongoing (starting June 25, 2020). We anticipate to complete recruitment by June 30, 2021 and complete the study by September 30, 2021.

**Trial registration:**

ClinicalTrials.gov protocol ID NCT04368676 (posted April 30, 2020).

**Full protocol:**

The full protocol is attached as an additional file, accessible from the Trials website (Additional file 1). In the interest in expediting dissemination of this material, the familiar formatting has been eliminated; this Letter serves as a summary of the key elements of the full protocol.

## Supplementary information

**Additional file 1.**

## Data Availability

Not applicable.

