# Supplementary material for: Breath Regulation and yogic Exercise An online Therapy for calm and Happiness (BREATH) for frontline hospital and long-term care home staff managing the COVID-19 pandemic: A structured summary of a study protocol for a feasibility study for a randomised controlled trial
Source: Trials. 2020 Jul 14;21:648. doi: 10.1186/s13063-020-04583-w (PMC7359429; doi:10.1186/s13063-020-04583-w)

**B**reath **R**egulation and yogic **E**xercise **A**n online **T**herapy for calm and **H**appiness (**BREATH**): an RCT for frontline hospital and long-term care home staff managing the COVID-19 pandemic

**Principal Investigator:** Dr. Akshya Vasudev

**Department:** Division of Geriatric Psychiatry, Department of Psychiatry

**Address:** Associate Professor of Psychiatry and Neuroscience, Western University

#A2-607, Victoria Hospital, London Health Sciences Centre, 800 Commissioners Road East, London, ON, Canada

**Email:** [akshya.vasudev@lhsc.on.ca](mailto:akshya.vasudev@lhsc.on.ca)

**Phone:** 519-685-8500 ext. 75504

**Website:** Geriatric Mood Disorders Lab (<http://publish.uwo.ca/~avasude2/>)

**Trial Registration info:** **NCT04368676**

**Funding source: PI Start-up funds**

Table of Contents

[Executive Summary 3](#_Toc38635931)

[Background 4](#_Toc38635932)

[Objectives 5](#_Toc38635933)

[Hypotheses 5](#_Toc38635934)

[Study Design 5](#_Toc38635935)

[Randomization 5](#_Toc38635936)

[Eligibility Criteria 6](#_Toc38635937)

[Inclusion Criteria 6](#_Toc38635938)

[Exclusion Criteria 6](#_Toc38635939)

[Study Outcomes 6](#_Toc38635940)

[Feasibility Outcomes 6](#_Toc38635941)

[Intervention Outcomes 6](#_Toc38635942)

[Insomnia 6](#_Toc38635943)

[Anxiety 6](#_Toc38635944)

[Depression 7](#_Toc38635945)

[Resilience 7](#_Toc38635946)

[Study Procedures 7](#_Toc38635947)

[Recruitment 7](#_Toc38635948)

[Screening 7](#_Toc38635949)

[Study Interventions 8](#_Toc38635950)

[Study Assessments 8](#_Toc38635951)

[Statistical Considerations 9](#_Toc38635952)

[Expected Results and Significance 10](#_Toc38635953)

[Limitations 10](#_Toc38635954)

[Adverse Events 11](#_Toc38635955)

[Study Personnel 11](#_Toc38635956)

[References 12](#_Toc38635957)

[Appendix 14](#_Toc38635958)

[Contact Information Form 14](#_Toc38635959)

[Screening Questionnaire 14](#_Toc38635960)

[Demographics Questionnaire 15](#_Toc38635961)

# Executive Summary

The COVID-19 pandemic continues to escalate as a global health threat, leading to devastating impacts on the healthcare system. Frontline healthcare workers are dealing with increased demands and fears for their own, as well as their family’s safety. Adding to the stress is the limited ability to maintain social connectedness as physical distancing measures are in place. We plan to conduct an open-label feasibility randomized controlled trial (RCT), comparing an online breath based yogic intervention called Sudarshan Kriya Yoga (SKY) (*n*=30) versus an online control mind-body intervention called the Health Enhancement Program (HEP) (*n*=30) in 60 frontline hospital and long-term care home staff managing the COVID-19 pandemic. Participants will be blinded to the treatment hypothesis while data analyst will be blinded to treatment allocation. Both SKY and HEP will be taught online in two phases in the first week followed by weekly reinforcement sessions for the following 4 weeks. Feasibility measures will be assessed as well as self-rated measures of insomnia, anxiety, depression, and resilience. We expect that it will be feasible to run an online randomized controlled trial of these two online interventions, SKY and HEP, in staff managing the COVID-19 pandemic, and that we will be able to remotely monitor safety and efficacy of these interventions.

# Background

Severe acute respiratory syndrome coronavirus 2 (SARS-CoV-2), the virus causing coronavirus disease 2019 (COVID-19), has rapidly spread. As of April 10, 2020, more than 1.6 million cases of COVID-19 and more than 100,000 deaths have been reported worldwide (1). This novel disease has led to an increased burden on the global healthcare system. Frontline healthcare staff managing COVID-19 patients are working long and repeated shifts. Besides suffering from exhaustion, their mental health has been severely challenged. The daily difficult ethical and moral decisions on how to provide care (2), as well as their own risk of contracting the illness and bringing it home remain their highest concerns. This has been evident in China and Italy, where staff have reported as high as 50% incidence of insomnia, stress, anxiety and depression, all likely affecting their decision-making capacity (3, 4).

The implementation of physical distancing measures has likely led to increased feelings of isolation in these staff, which is expected to lead to an exacerbation of mental health issues (5). The current inability to provide a face-to-face psychosocial interaction makes supporting these frontline staff more challenging. While a number of online support mechanisms have been made available to these staff (6), it is likely that they are either unaware of them and/or lack the motivation to use them. Additionally, available online resources are not interactive or inherently inspirational, and do not provide a sense of community or the opportunity for social interaction. Hence, it can be expected that frontline healthcare staff will remain highly prone to mental illness, and, unable to build their resilience during long and stressful shift work.

Sudarshan Kriya Yoga (SKY) is a standardized, easy to learn breath based yogic intervention program consisting of breath control techniques followed by a timed rhythmic breathing practice consisting of fast, medium and slow breaths. SKY, once learnt, takes between 10-25 minutes to practice daily at home. The SKY program has until recently been taught in community groups by the charitable and humanitarian organization, the Art of Living Foundation (AOLF), to at least 6 million people across 155 countries. Research on SKY conducted by independent scientists including ourselves has demonstrated large effect sizes in clinical samples of anxiety, depression as well as PTSD (7). While the biological basis of such a response is actively being explored, SKY has been shown to induce a parasympathetic stress alleviating response, reduction of central sympathetic drive, an improvement in heart rate variability and the hypothalamic pituitary adrenal axis driven immune response as well as anti-apoptotic and anti-oxidant effects (8, 9).

Due to the COVID-19 pandemic, AOLF has recently been able to successfully deliver SKY via an online platform across Asia and North America. However, it is currently unknown if a) an online version of SKY can be delivered to frontline healthcare staff and b) if SKY improves measures of insomnia, anxiety, depression and resilience in such staff. The aim of this study is to assess the effects on an online SKY program individualized for frontline hospital and long-term care home staff who are managing the COVID-19 pandemic. As this will be a pilot study, key feasibility measures, including recruitment, retention and applicability will be collected with the aim of using these for further designing a large multicentric RCT in the future.

An active control arm, the Health Enhancement Program (HEP), will be used to control for several non-specific factors found in a SKY group, including: group support and morale, behavioural action, reduction of stigma, facilitator attention, treatment duration, and time spent on at-home practice. HEP will consist of time-matched online self-paced modules, comprised of de-stressing guided exercises such as gentle stretch and yoga asanas and progressive muscle relaxation. In addition, mindfulness-based meditation sessions delivered by a senior psychiatry resident and backed up by mental health social workers will be included. Either of the online interventions, SKY or HEP, may help improve the mental health of frontline healthcare staff in an easily accessible and interactive format.

# Objectives

**Objective 1:** To determine if it is feasible to conduct an RCT of online SKY for frontline hospital and long-term care home staff under the constraints imposed by the COVID-19 pandemic and need for remote trial monitoring.

**Objective 2:** To assess whether online versions of SKY and/or HEP result in improvement in self-rated measures of insomnia, anxiety, depression, and resilience.

# Hypotheses

**Hypothesis 1:** It will be feasible to conduct an RCT of online SKY for frontline hospital and long-term care home staff under the constraints imposed by the COVID-19 pandemic and need for remote trial monitoring.

**Hypothesis 2:** Compared to an online Health Enhancement Program (HEP) active control, an online version of SKY will offer more meaningful improvement in self-rated measures of insomnia, anxiety, depression, and resilience.

# Study Design

This study will be an open-label feasibility RCT comparing 5-week online versions of SKY and HEP in 60 participants randomized equally to one of the two groups. Participants will be asked to complete brief, online self-rated questionnaires at weeks 0, 3, and 5. We will offer 3 cohorts with a mean of 20 participants each over spring and summer of 2020. All components of the study will be delivered in an online format using REDCap and Cisco WebEx secure software. Randomization and its concealment will be achieved through REDCap, an electronic data capture system offered by the Lawson Health Research Institute and freely available to Lawson’s research staff. Group allocation will be masked during analysis.

# Randomization

Participants will be randomized after they have electronically signed the consent form and the research staff have confirmed eligibility. We will use REDCap to perform randomization as well as allocation concealment. REDCap is widely used by health researchers worldwide to significantly reduce data entry and study management errors to improve data fidelity. All study participants will be blinded to the study hypotheses so as to prevent any expectation bias.

# Eligibility Criteria

## Inclusion Criteria

1. Participants will be frontline hospital or long-term care home staff involved in the management of COVID-19 patients in London, ON, Canada.
2. Participants will be aged 18 to 70
3. Participants will be willing and able to attend, via WebEx software, the two phases of SKY or HEP, as well as weekly follow-up sessions
4. Have sufficient hearing to follow verbal instructions.
5. Have an adequate understanding of English.
6. Able to sit without physical discomfort for 60 minutes.
7. Not pregnant and willing to remain not pregnant for the duration of the study.

## Exclusion Criteria

1. Inability to independently provide informed consent.
2. Current suicidality as assessed by the suicide item of the Patient Health Questionnaire-9 scale.
3. History of bipolar disorder.
4. History of chronic PTSD.
5. History of schizophrenia or schizoaffective disorder
6. Currently practice any type of formal meditation, mindfulness, or breathing techniques regularly (greater than 3 times per week).

# Study Outcomes

## Feasibility Outcomes

The following feasibility outcomes will be measured: (1) rate of participant recruitment, (2) rate of retention, (3) completeness of data entry, (4) cost of interventions, and (5) unexpected costs. Such measures will be collected on a daily basis through-out the study and tabulated 5 weeks later at the end of the study.

## Intervention Outcomes

### Insomnia

The Athens Insomnia Scale is an 8-item, self-rated measure of the extent of sleep difficulties over the last 30 days with each item representing a different aspect of sleep (e.g. “Overall Quality of Sleep”). Ratings are from 0 to 4, with 0 being no difficulty with the item and 4 being the most difficulty with the item. Higher overall scores on this scale indicate higher difficulties with sleep (10).

### Anxiety

The Generalized Anxiety Disorder 7-item scale (each scored 0–3) is a self-rated measure of anxiety and has been validated for the diagnosis of Generalized Anxiety Disorder in the adult population. Scores range from 0 to 21. Higher scores indicate greater anxiety symptoms (5–9, mild anxiety; 10–14, moderate anxiety; 15–21 severe anxiety) (11).

### Depression

The Patient Health Questionnaire (PHQ-9) is a 9-item, self-rated measure of depression which has been validated for screening a major depressive episode in adults. Total scores indicate various levels of depression: 0–4, no depression; 5–9, mild depression; 10–14, moderate depression; 15–19, moderately severe depression; 20–27, severe depression (12, 13).

### Resilience

The Connor-Davidson Resilience Scale (CD-RISC) is a brief self-rated assessment tool that comprises of 10 items, each rated on a 5-point Likert scale (0 = not true at all to 4 = true nearly all the time). Higher scores reflect greater resilience, which is a measure of one’s ability to cope with stress. The scale demonstrated strong reliability and validity and has been studied in a variety of populations including medical personnel, nurses, social workers and physicians (14).

# Study Procedures

## Recruitment

Participants will include frontline hospital and long-term care home staff (*n*=60) that are involved in the management of COVID-19 patients in London, ON, Canada. Feedback from physicians and nurses working in the ICUs and Emergency Room at LHSC shows that they are currently under immense stress and hence they will be targeted first. Additional staff will be targeted during the study as the pandemic unfolds and additional staff are expected to be redeployed. The study will recruit via emails and posters, as well as through established connections between the study PI, Co-investigators and LHSC staff. Posters will be placed online on the websites of the study’s investigator as well as websites and social media sites of relevant organizations. Online posters will indicate that individuals should not share or comment on posters to protect their identity. This study will leverage the networks of the PI and co-investigators to actively look towards recruitment through emails and posters in these departments. Directors of Care of long-term care homes in London, ON Canada will be contacted via email to recruit potential participants. Using these methods, the study is expected to recruit approximately 5 participants per week.

## Screening

Interested participants will be provided with an electronic Letter of Information (LOI) to review and consider participation. If interested participants have questions after reading the LOI, they will be offered a secure video chat with the study Research Assistant via Cisco WebEx to further explain the study and answer questions. Potential participants who electronically sign the LOI and submit via REDCap will be immediately provided with the contact information form requesting contact information including email address and phone number (once LOI is submitted the form will be automatically generated in a new page). Following completion of the contact information form participants will next receive a page with the screening questionnaire. REDCap software will produce an immediate email alert once the screening questionnaire has been completed. If needed the PI, or a covering psychiatrist investigator (JS) if the PI is not available, will contact the participant for diagnostic clarification/suicide risk assessment as soon as possible and will additionally provide support as needed.

## Study Interventions

Both the SKY and HEP programs will be delivered using the Hospital approved Cisco WebEx platform. The online version of SKY will be delivered by at least one certified Canadian SKY teacher, with at least one back up teacher at all times, under the supervision of Ms. Ronnie Newman, Director of Research and Health Promotion, Art of Living Foundation, USA. The online version of SKY for healthcare workers has a total duration of 3 hours. Phase I will consist of 5 self-paced online modules of 4-10 minutes each to learn the breath control techniques. Participants will be sent an online survey in REDCap requesting that they self-confirm completion of the Phase I modules. In Phase II, 2 interactive online sessions of 1 hour each will be held on consecutive days with a certified SKY teacher, during which participants will learn the fast, medium and slow breaths. For ease of scheduling, multiple time windows will be offered for Phase II. There will be at least one back up teacher at all times. Both Phase I and II will be completed in the first week. The active control arm, HEP, will consist of time-matched online self-paced modules for Phase I, comprised of de-stressing guided exercises such as gentle stretch and yoga asanas and progressive muscle relaxation. Phase II will consist of mindfulness-based meditation sessions delivered by Dr. Paris Lai, co-investigator and senior psychiatry resident, and backed up by Ms. Victoria Mills and Ms. Kasha Herba, MSWs, mental health social workers. They all have previous research experience in our Geriatric Mood Disorders Lab. Weekly follow up sessions will be offered to all recruited participants for 30 minutes each for the subsequent 4 weeks in both study arms. HEP teachers who are hospital staff will be emailed an anticipated attendance list including first names of participants in each group. In the event that two participants in a group have the same first name the first initial of the last name will also be used. SKY teachers will be sent a similar list by email via the hospital secure file share service. Facilitators of HEP and SKY will be requested to complete an attendance and comments list in REDCap following each session.

## Study Assessments

| **Assessment** | |  |  |  | **Date of administration** | | | |
| --- | --- | --- | --- | --- | --- | --- | --- | --- |
| **Name of Assessment** | **Variables** | **Screening** | **Week 0** | **Week 1** | **Week 2** | **Week 3** | **Week 4** | **Week 5** |
| Informed consent | Consent | X |  |  |  |  |  |  |
| Contact information form | Name, phone number, e-mail address | X |  |  |  |  |  |  |
| Screening questionnaire | Eligibility | X |  |  |  |  |  |  |
| Demographics questionnaire | Age, gender, past year substance use, profession, religious and spiritual affiliation, frequency of religious practice, previous history of mental health disorders |  | X |  |  |  |  |  |
| AIRS | Insomnia |  | X |  |  | X |  | X |
| GAD-7 | Anxiety |  | X |  |  | X |  | X |
| PHQ-9 | Depression |  | X |  |  | X |  | X |
| CD-RISC-10 | Resilience |  | X |  |  | X |  | X |
| Side effects week 1 | Adverse events and attendance |  |  | X |  |  |  |  |
| Side effects for follow-ups | Adverse events and attendance |  |  |  | X | X | X | X |

Abbreviations: Athens Insomnia Rating Scale (AIRS), Generalized Anxiety Disorder-7 scale (GAD-7), Patient Health Questionnaire-9 scale (PHQ-9), and Connor-Davidson Resilience Scale-10 (CD-RISC-10).

Once enrolled, the following demographic variables will be collected: age, gender (females have been found to be at higher risk of mental health disorders in China), profession (nurses have been found to be at higher risk), religious and spiritual affiliation, frequency of religious practice, previous history of mental health disorders, self-reported current alcohol and substance use. Current possible alcohol-use disorder will be screened using the National Institute on Alcohol Abuse and Alcoholism screening question which has a sensitivity of 82% and specificity of 79% (15). A single screening test for substance use in primary care will be used to screen for other possible substance use problems providing a sensitivity of 100% and specificity of 74% (16).

The following well validated self-rated scales will be administered online: Athens Insomnia Rating Scale (AIRS, with cut off of ≥ 8 for diagnosing sleep disorder), Generalized Anxiety Disorder-7 scale (GAD-7, with cut off of ≥ 10 for diagnosing anxiety disorder), Patient Health Questionnaire-9 scale (PHQ-9, with cut off of ≥ 10 for diagnosing depressive disorder), and Connor-Davidson Resilience Scale-10 (CD-RISC-10, U.S. general population mean = 32.1 and standard deviation = 5.8). We will collect qualitative comments for open feedback about the interventions, monitor for signs of severe mental distress and potential suicidality using both REDCap and WebEx’s secure feedback options.

All questionnaires will be administered at week 0, week 3, and week 5 by sending an email link directing participants to REDCap surveys. If a participant withdraws from the intervention for any reason, the reason for drop out will be collected by a WebEx meeting or telephone call and they will be invited to still complete any of the outcome measures that they are able and willing to complete. We will offer 3 cohorts with a mean of 20 participants each over spring and summer of 2020. Randomization and its concealment will be achieved through REDCap and groups will be masked during analysis. See appendix for details of contact information form and screening and demographics questionnaires.

# Statistical Considerations

All analysis will be conducted in an intention-to-treat (ITT) approach with the last observation carried forward (LOCF). A Multivariate Analysis of Variance (MANOVA) will be conducted to detect differences between the two interventions, i.e. SKY and HEP as the independent variables and scores on AIRS, GAD-7, PHQ-9, and CD-RISC-10 scale as the dependent variables. Multivariate F value (Wilks' λ or Hotelling's trace or Pillai's trace) will be used with a statistical significance set at p < 0.05. Demographic measures will be collated and percentage or means calculated as appropriate. Sample size estimates for pilot feasibility studies are advised not to be a priori attempted and there is no consensus on what a pilot study sample size should be (17). However, previous studies in samples of depression, anxiety and PTSD who have undergone SKY intervention demonstrated a large effect size (F of 0.4 and higher). Thus, a sample size estimate can be conducted for a repeated measures MANOVA with two groups (SKY vs. HEP) and 3 repeated measurements (week 0, week 3, and week 5) in G*Power using an alpha of 0.05 and power of 0.95 (18). Assuming that there is no correlation between repeated measurements and the covariates also leads to a more conservative assessment for an effect size. Thus, based on the aforementioned assumptions, the desired sample size is 36. Accounting for a possible 40% attrition rate, the desired sample size is 60.

Data analysis will be carried out using IBM SPSS ® (v23) (19). Data will be assessed for normality using either the Kolmogorov-Smirnov or the Shapiro-Wilk test depending on the final sample size. Missing data will be imputed using multiple imputation with the Expectation-Maximization (EM) algorithm (20, 21). Statistical analyses will be run twice: first, according to the ITT analysis, and second, only with participants who had completed the study (per protocol analysis [PP]). In both the ITT and PP analyses, the differences in insomnia, anxiety, depression and resiliency outcomes between groups at week 0, week 3, and week 5 will be analyzed using a two (group) by three (time) two-way repeated measures MANOVA to test the time and group interactions term (i.e. time * group). The analyses will be controlled for profession in the ITT analysis and hours worked per week in the PP analysis.

# Expected Results and Significance

We expect that it will be feasible to conduct an RCT of online SKY versus HEP intervention for frontline healthcare staff and long term care workers in London, Ontario, Canada. It will also be feasible to minimise risk to the PI, co-investigators, and research staff by collecting and monitoring all data remotely. Based on strong prior evidence in similar populations, we do expect that compared to an online HEP active control group, those who complete the online SKY intervention will have more meaningful improvements in self-rated measures of insomnia, anxiety, depression and resilience. Overall, this study is timely as frontline hospital and long-term care home staff managing COVID-19 patients are faced with an extremely high risk of mental illness and reduced well-being. The data from this study will be right in-time and pivotal to inform if online innovative group-based mind-body interventions , such as SKY and/or HEP, are of benefit to improve mental wellness and resilience.

# Limitations

Given the unique challenges to our healthcare system posed by the COVID-19 pandemic, participants might find it difficult to find the time to attend the 3 hours of initial training. Additionally, they might themselves contract the illness while at work and henceforth might need to undergo isolation. Hence, there remains a high risk of drop-out. For these reasons we will offer the intervention staggered, out of regular hours, at times that are most convenient for participants. This study will also leverage the networks of the PI and co-investigators to actively seek recruitment through word of mouth and putting up posters in key clinical departments. Nevertheless, we have accounted for a possible high attrition rate in our sample size estimates.

Given the current 14-day isolation period for staff who might have been exposed to COVID-19 and are under quarantine, this period offers a unique opportunity to still participate in the study and learn the skills from the trial interventions. Other unique aspects of our study, compared to current standard of care of receiving emails or links of mental health resources, is that both arms have been designed to improve social connectedness while maintaining social distancing using videoconferencing technology. These advantages will be highlighted during the recruitment process. Lastly, by keeping a reasonably low number of self-rated as well as brief questionnaires, we hope to collect essential outcome data and yet not have respondent fatigue. Overall, this study is innovative and offers strong evidence-based interventions using safe, and likely effective, online platforms. The results generated will offer us the ability to move quickly towards larger provincial and federal grant opportunities for a subsequent large scale multicentric RCT.

# Adverse Events

During the enrollment process participants will be instructed to contact the research team via WebEx/telephone if they are having deterioration in their mental health. WebEx contact information and phone numbers for the research team will provided, as well as the phone number for the Canadian Mental Health Association help line, which they may access 24/7. Participants will be asked to report any side effects via REDCap at weeks 1, 2, 3, 4, and 5. Reported side effects will be reviewed by the PI or if the PI is unavailable the back-up Co-I psychiatrist (JS) within 24 hours of reporting. Standard of care will be unaffected and only capable and consenting patients will be recruited into the study.

# Study Personnel

| **Name** | **Role in Study** | **E-mail** |
| --- | --- | --- |
| Akshya Vasudev | Principal Investigator | [akshya.vasudev@lhsc.on.ca](mailto:akshya.vasudev@lhsc.on.ca) |
| Imants Baruss | Co-Investigator | [baruss@uwo.ca](mailto:baruss@uwo.ca) |
| Ka Sing Paris Lai | Co-Investigator | [paris.lai@lhsc.on.ca](mailto:paris.lai@lhsc.on.ca) |
| Cheryl Forchuk | Co-Investigator | [cforchuk@uwo.ca](mailto:cforchuk@uwo.ca) |
| Javeed Sukhera | Co-Investigator | [javeed.sukhera@lhsc.on.ca](mailto:javeed.sukhera@lhsc.on.ca) |
| Amer Burhan | Co-Investigator | [amer.burhan@sjhc.london.on.ca](mailto:amer.burhan@sjhc.london.on.ca) |
| Emily Ionson | Research Coordinator | [emily.ionson@lhsc.on.ca](mailto:emily.ionson@lhsc.on.ca) |
| Christine Watt | Research Assistant | [christine.watt@lhsc.on.ca](mailto:christine.watt@lhsc.on.ca) |

# References

1. World Health Organization. Coronavirus disease 2019 (COVID-19) Situation Report

– 76 2020 [updated April 5, 2020. Available from: https://www.who.int/docs/default-source/coronaviruse/situation-reports/20200405-sitrep-76-covid-19.pdf?sfvrsn=6ecf0977_4.

2. Rosenbaum L. Facing Covid-19 in Italy — Ethics, Logistics, and Therapeutics on the Epidemic’s Front Line. New England Journal of Medicine. 2020.

3. Lai J, Ma S, Wang Y, Cai Z, Hu J, Wei N, et al. Factors Associated With Mental Health Outcomes Among Health Care Workers Exposed to Coronavirus Disease 2019. JAMA Network Open. 2020;3(3):e203976-e.

4. Mortillaro N. 'I've never been so stressed': Health-care workers in COVID-19 battle face PTSD, mental health issues. CBC News. 2020.

5. Rohde N, D'Ambrosio C, Tang K, Rao P. Estimating the mental health effects of social isolation. Applied research in quality of life. 2016;1(3):853-69.

6. Canada CS. COVID-19 Resources: Mental Health Commission of Canada; 2020 [Available from: <https://www.crisisservicescanada.ca/en/covid-19-resources/>.

7. Descilo T, Vedamurtachar A, Gerbarg PL, Nagaraja D, Gangadhar BN, Damodaran B, et al. Effects of a yoga breath intervention alone and in combination with an exposure therapy for post-traumatic stress disorder and depression in survivors of the 2004 South-East Asia tsunami. Acta Psychiatr Scand. 2010;121(4):289-300.

8. Brown RP, Gerbarg PL. Sudarshan Kriya yogic breathing in the treatment of stress, anxiety, and depression: part I-neurophysiologic model. J Altern Complement Med. 2005;11(1):189-201.

9. Brown RP, Gerbarg PL. Sudarshan Kriya Yogic breathing in the treatment of stress, anxiety, and depression. Part II--clinical applications and guidelines. J Altern Complement Med. 2005;11(4):711-7.

10. Soldatos CR, Dikeos DG, Paparrigopoulos TJ. Athens Insomnia Scale: validation of an instrument based on ICD-10 criteria. J Psychosom Res. 2000;48(6):555-60.

11. Spitzer RL, Kroenke K, Williams JB, Lowe B. A brief measure for assessing generalized anxiety disorder: the GAD-7. Arch Intern Med. 2006;166(10):1092-7.

12. Kroenke K, Spitzer RL, Williams JB. The PHQ-9: validity of a brief depression severity measure. J Gen Intern Med. 2001;16(9):606-13.

13. Kroenke K, Spitzer RL, Williams JB, Lowe B. The Patient Health Questionnaire Somatic, Anxiety, and Depressive Symptom Scales: a systematic review. Gen Hosp Psychiatry. 2010;32(4):345-59.

14. Connor KM, Davidson JR. Development of a new resilience scale: the Connor-Davidson Resilience Scale (CD-RISC). Depress Anxiety. 2003;18(2):76-82.

15. Smith PC, Schmidt SM, Allensworth-Davies D, Saitz R. Primary care validation of a single-question alcohol screening test. J Gen Intern Med. 2009;24(7):783-8.

16. Smith PC, Schmidt SM, Allensworth-Davies D, Saitz R. A single-question screening test for drug use in primary care. Arch Intern Med. 2010;170(13):1155-60.

17. Teare MD, Dimairo M, Shephard N, Hayman A, Whitehead A, Walters SJ. Sample size requirements to estimate key design parameters from external pilot randomised controlled trials: a simulation study. Trials. 2014;15(1):264.

18. Faul F, Erdfelder E, Buchner A, Lang A-G. G*Power Version 3.1.7 [computer software] Uiversität Kiel, Germany2013 [Available from: <http://www.psycho.uni-duesseldorf.de/abteilungen/aap/gpower3/download-and-register>.

19. IBM Corp. IBM SPSS Statistics for Windows. 23.0 ed. Armonk, NY: IBM Corp.; 2015.

20. Dong Y, Peng C. Principled missing data methods for researchers. SpringerPlus; 2013. p. 222

21. Peng C, Harwell M, Liou S, Ehman L. Advances in missing data methods and implications for educational research. Real Data Anal.; 2006. p. 31-78.

# Appendix

## Contact Information Form


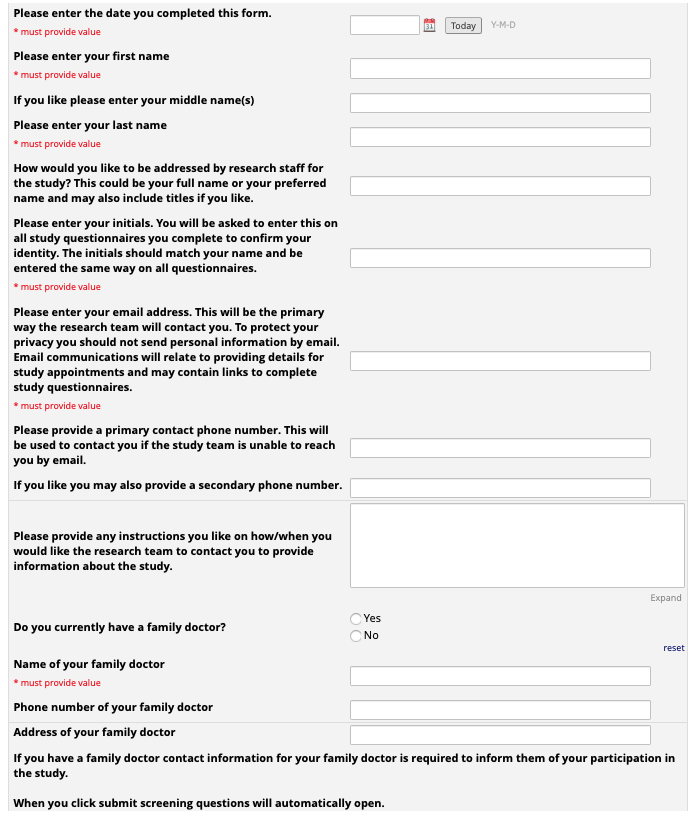


## Screening Questionnaire

##
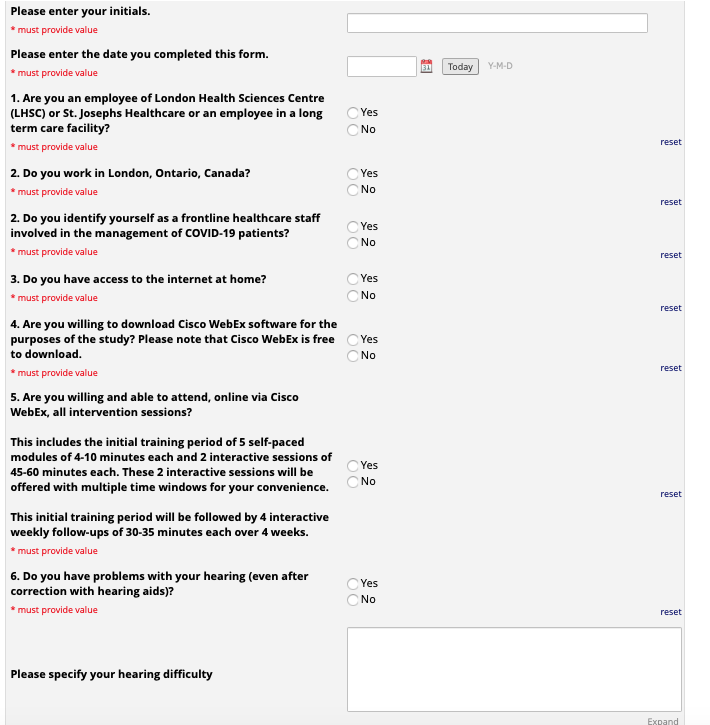


##
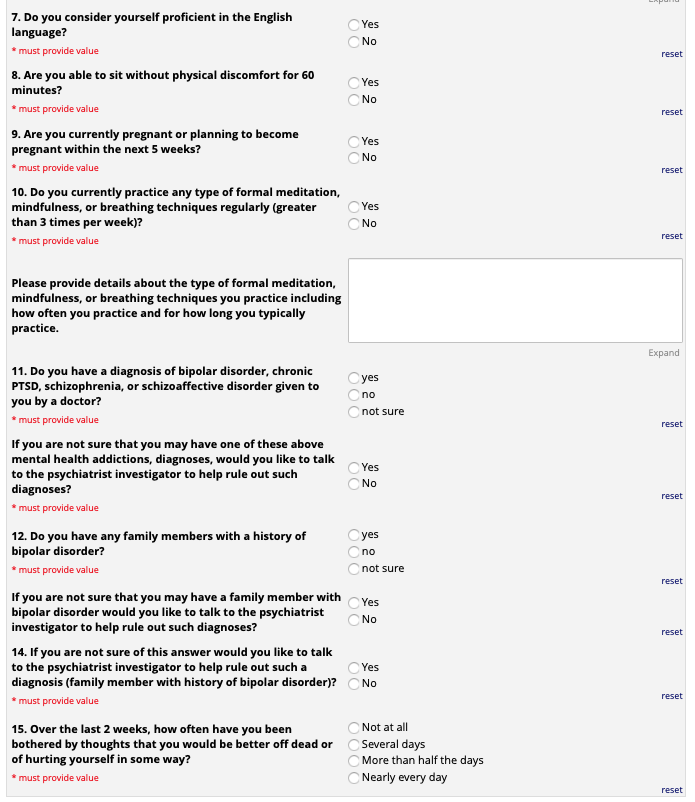


## Demographics Questionnaire


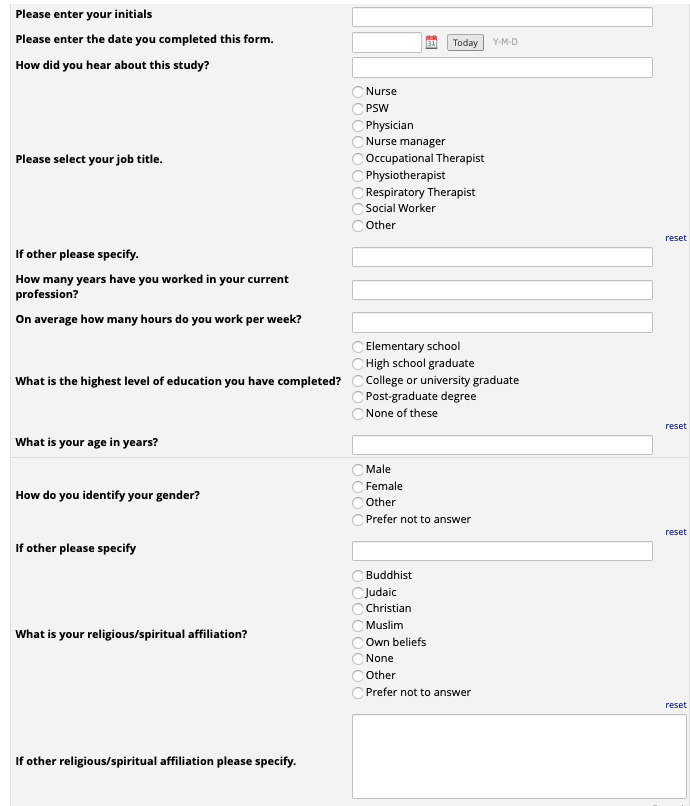


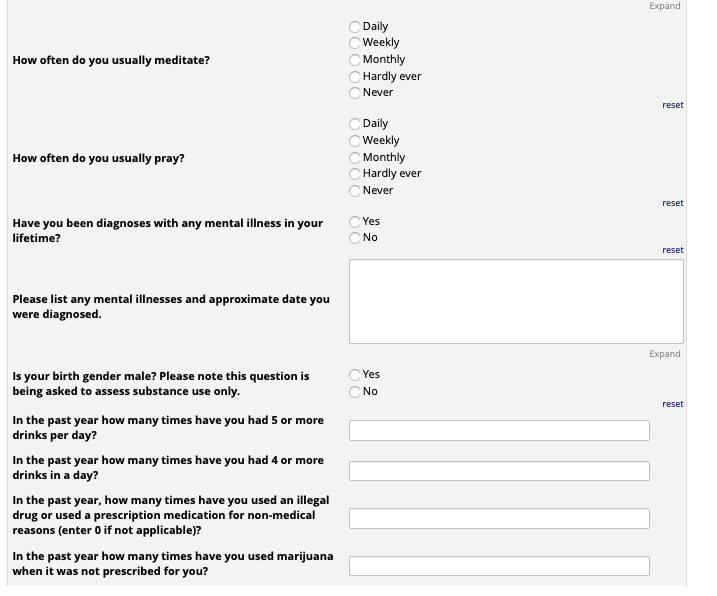

Supplement: Supplementary file 1 — Additional file 1. [file 13063_2020_4583_MOESM1_ESM.docx]
